# ‘A race against time’: a photovoice study on priorities for quality of injury care with patients, health workers and policymakers in four low- and middle-income countries

**DOI:** 10.1136/bmjgh-2026-024260

**Published:** 2026-08-20

**Authors:** The Equi-Injury Group, Lucia D’Ambruoso, Lucia D’Ambruoso

**Keywords:** Injury

## Abstract

**Introduction:**

The Lancet Commission on High Quality Health Systems called for quality measures grounded in user experience. Studies of how stakeholders interpret and prioritise these frameworks are rare for injury care in low- and middle-income countries (LMICs).

**Objectives:**

The objective was to identify how patients, healthcare providers and policymakers in four LMICs prioritise quality of care after injury using Institute of Medicine (IoM) quality domains (safety, effectiveness, patient-centredness, timeliness and equity).

**Methods:**

We conducted a Photovoice study across eight urban and rural sites in Ghana, Pakistan, Rwanda and South Africa. Focus group discussions (n=39) convened patients/community members, healthcare providers and policymakers (n=116), supplemented by policymaker interviews (n=6). Participants photographed aspects of injury care, developed captions and used deliberative dialogue to rank these against the IoM domains.

**Results:**

Stakeholders prioritised timeliness, safety and effectiveness, with rankings varying by local system capability. In settings with significant trauma system gaps (Ghana, Pakistan), timeliness ranked highest: immediate survival, getting to care quickly across transport, referral and facility processes. Safety was also emphasised: avoiding additional harm from unsafe and unreliable environments and resources. In settings with more established access to basic emergency care (Rwanda, South Africa), priorities shifted towards effectiveness, understood in terms of clinical, functional and psychosocial recovery. Across all contexts, equity (fair access and financial protection) was interpreted as foundational rather than as a discrete domain, shaping delays, access, treatment continuity and catastrophic costs. Overall, quality was considered cumulative and relational: early failures in care pathways amplified avoidable downstream complications including disability and impoverishment.

**Conclusions:**

Stakeholders shared systems understandings of injury care quality, with priorities varying by local capabilities. Timeliness and safety were seen as necessary for survival, enabling effectiveness in clinical and recovery outcomes, while equity operated across care pathways. The findings support aligning emergency quality indicators and improvement strategies with context-specific stakeholder priorities.

**WHAT IS ALREADY KNOWN ON THIS TOPIC**

- Injuries cause disproportionate death and disability in low- and middle-income countries, making quality of injury care a salient health system priority.
- Frameworks such as the Institute of Medicine quality domains guide improvement efforts.
- Little is known about how patients, providers and policymakers themselves interpret and prioritise these frameworks and their components.

**WHAT THIS STUDY ADDS**

- Across eight sites in Ghana, Pakistan, Rwanda and South Africa, stakeholders framed quality as cumulative across the care pathway: early failures amplified downstream complications, disability and costs.
- Priorities varied with system capabilities: timeliness and safety predominated where trauma system gaps were acute; effectiveness predominated where basic emergency access was more established.
- Across contexts, equity was positioned as foundational, shaping more proximate and visible constraints and priorities related to access, delays and financial protection.

**HOW THIS STUDY MIGHT AFFECT RESEARCH, PRACTICE OR POLICY**

- The findings support integrated prehospital, facility-based and financial protection reforms calibrated to local system maturity.
- Universal quality indicators should be complemented by contextually sensitive measures reflecting local features including emergency care systems development.

## Introduction

 Injuries remain a leading cause of death and disability in low- and middle-income countries (LMICs).^1^ Improving quality of care (QoC) for injured people is a central strategic priority.^2^ Global frameworks such as the Institute of Medicine (IoM) quality domains provide benchmarks to inform policy, planning and quality improvement.^3^ The Lancet Commission on High Quality Health Systems called for quality measures grounded in user experience, yet this has rarely been applied to injury care in LMICs.^4^

Prioritisation exercises offer structured approaches to identify aspects of quality relevant to different groups.^5^ Combined with participatory methods, they enable participants to express contextually grounded priorities, depicted through images and narratives and collectively validated through consensus.^6 7^ Such approaches enrich discussions of QoC, highlighting contextual, relational and experiential dimensions often under-represented in standard frameworks and metrics.

This study was conducted across Ghana, Pakistan, Rwanda and South Africa, as part of the National Institute for Health and Care Research Equi-Injury programme.^8^ The partner settings in Equi-Injury were selected to capture diverse injury burdens, health systems organisation and financing and emergency system maturity in sub-Saharan Africa and South Asia. Across these settings, injuries account for substantial mortality and disability, and health systems face persistent challenges in timely access and emergency care capacity.^9 10^ Injury care in LMICs also receives disproportionately low investment relative to its disease burden,^2 10^ shaping how injury care is operationalised. Despite differences in health system organisation and financing including high out-of-pocket (OOP) expenditure in Pakistan,^11^ community-based health insurance in Rwanda,^12^ a dual public–private system in South Africa^13^ and social health insurance in Ghana with notable gaps for rehabilitation,^14^ questions of quality remain central to improving outcomes for injured people.^9^

We aimed to identify how patients and community members, healthcare providers and policymakers in four LMIC settings interpreted and prioritised the IoM quality domains for injury care. We report the dimensions ranked as most important, the consensus and divergence between groups and the implications for contextually sensitive quality improvement. Photovoice was selected as an approach suited to participants with varying levels of formal education, able to surface contextual, visual and relational dimensions of care and compatible with deliberative prioritisation.

## Methods

### Study design and setting

We conducted a Photovoice study (September–December 2024). Focus group discussions were incorporated for orientation and prioritisation with deliberative prioritisation as the analytical focus. Focus groups enabled collective deliberation and consensus building; photographs and captions surfaced lived and visual dimensions not easily captured in text. In Pakistan, interviews with policymakers were performed, and we used post hoc aggregation where convening a group was not feasible. Eight sites were selected to represent diverse health systems, including urban tertiary centres and rural district catchment areas in each setting (table 1).

**Table 1:** Comparative characteristics of study settings in Ghana, Pakistan, Rwanda and South Africa

Country characteristics	Injury burden and context	Governance and policy	Engagement	Systems/data	Study sites
Ghana Pop: ~34 million Income: Lower-middle^38^	~8% of deaths; 7% of DALYs.^39^ National Health Insurance operational since 2003, but high OOP limits access.^40^ Long-standing challenges in investment and implementation of injury care systems.^14^	Moderately decentralised via district health systems and assemblies. Injury is recognised in national NCD and road safety policies, but implementation is weak.	District Health Committees; traditional leadership structures.	Despite improvements, implementation of prehospital and emergency care remains weak due to systemic underinvestment and resource constraints.^41^ Ambulance services coverage has improved despite challenges.^42^	Urban: Tamale Rural: Bekwai
Pakistan Pop: ~241 million Income: Lower-middle^38^	~7% of deaths.^39^ Rapid urbanisation with high demand/poor supply of critical care. High reliance on the private sector and OOP payments; national health coverage is currently rolling out.^11^	Decentralised federal system (post-18th Amendment), provinces lead healthcare delivery. National Injury Prevention Policy, however, data and implementation incomplete.	Union Councils, Panchayats, and emerging digital forums.	Fragmented injury care; no single National Trauma System. Federal constitutional responsibility for health information but difficulties with the national injury registry under devolution.^43^	Urban: Karachi (Aga Khan University Hospital; Jinnah Post Graduate Medical Centre) Rural: Sehwan, Thatta
Rwanda Pop: ~13 million Income: Low^44^	~9% of deaths; 10% of DALYs.^23^ High burden linked to social challenges post-1994 genocide against the Tutsi. High community-based insurance coverage and OOP costs. Strong PHC, but tertiary/surgical capacity limited.^45^	Strong top-down coordination, decentralised local delivery. Injury prevention is integrated into the national NCD plan and road safety policy.	Extensive decentralised planning units combined with strong central control; expansion of PHC.	PHC is robust, however, tertiary and surgical trauma capacity remains limited. Injury data integration is improving, though gaps persist in measuring prehospital and long-term functional outcomes.^12^	Urban: Musanze/Kigali Rural: Kibogora/Kibungo
South Africa Pop: ~61 million Income: Upper-middle^46^	Burden: ~13% of deaths, IPV and RTI.^47^ High IPV and substance abuse. Dual system: majority of the population is reliant on public sector services, while resources are concentrated in the private sector reflecting entrenched inequalities.^13^	Mixed national-provincial-district system; traditional authorities recognised. Addressed in NCD strategy and violence/trauma policy.	Clinic Committees, District Health Councils, and traditional councils.	Two-tier system resource-rich private sector serves insured minority, public sector struggles with high/complex patient volumes and inequitable access, complicating national trauma governance.^13^	Urban: Khayelitsha (Western Cape) Rural: Ngqamakhwe (Eastern Cape)

DALYs, disability-adjusted life-years; IPV, interpersonal violence; NCD, non-communicable disease; OOP, out-of-pocket; PHC, primary healthcare; Pop, population; RTI, road traffic injury.

### Participants and recruitment

Stakeholder groups were recruited to represent: (a) community members and patients (individuals who had accessed inpatient injury care >12 hours or had moderate-to-severe injuries but did not access care); (b) healthcare providers (front-line staff involved in injury care) and (c) policymakers (civil servants with injury care policy responsibilities). Participants were drawn from Equi-Injury stakeholder forums supported by community engagement involvement structures.^15^ Within these, community and patient participants were identified through community gatekeepers, hospital outreach contacts, primary healthcare (PHC) networks, with iterative outreach to ensure inclusion of those who had not accessed care. We aimed for 5–6 participants per group, convening separate urban and rural groups for patients/community members and healthcare providers, supplemented by policymaker workshops. A total of 116 participants were engaged across 39 workshops.

### Data collection

Orientation (focus group discussion (FGD 1): Participants’ knowledge and experiences of injury care quality were elicited in open discussion. Facilitators then introduced IoM quality domains: safety, effectiveness, patient-centredness, timeliness and equity (online supplemental material 1). Domains were used as sensitising tools through which participants shared local meanings, experiences and perspectives. The efficiency domain was not included,^8^ because issues such as waste, duplication and value-for-money were considered reflective of system financing and organisation, and not comparable across stakeholders/settings. Efficiency-related concerns were nevertheless captured through other domains. Orientation to Photovoice was also provided: guiding participants to capture aspects of QoC, select and caption key images and appreciate the ethical implications and requirements of the technique.

Prioritisation (FGD 2): Participants reconvened 7–10 days later to present images and captions, map these to IoM domains, deliberate on and rank priorities. Facilitators projected selected photographs to prompt group deliberation and support consensus-building on priorities and how they reflected the IoM domains. In Pakistan, FGDs with policymakers were not feasible and interviews (n=6) were conducted using the same topic guide, with rankings aggregated. Researchers completed Rapid Research Evaluation and Appraisal Lab (RREAL) sheets for each data collection point to summarise substantive material and procedural reflections.^16^

### Data analysis

A frequency of endorsement (FoE) was calculated from the domain rankings for each setting and stakeholder group as a descriptive summary of how priorities were distributed within-groups and for cross-site comparison. The FoE was the number of participants selecting a domain as a proportion of the total number of participants in that group (range 0.00–1.00).

FoE scores were triangulated with qualitative analyses. Audio recordings were transcribed and translated into English. Drawing on transcripts and RREAL sheets, a coding matrix was developed based on the IoM domains and supplemented with emergent subthemes. Analysis was conducted in NVivo V.12.

### Ethical considerations

Ethical approval was obtained from Ghana Health Service Ethics Review Committee (GHS-ERC 014/09/22); Aga Khan University Ethics Review Committee (2022-7372-23339); Rwanda National Ethics Committee (85/RNEC/2023; 14/RNEC/2024 (renewal)) and Stellenbosch University Health Research Ethics Committee (N22/07/079). Written informed consent was obtained from all participants, and separately for subjects of images where necessary.

Deliberative prioritisation supported coproduction,^17^ ensuring participants selected, narrated and collectively interpreted images and agreed final priority rankings. Facilitators monitored participant well-being during data collection, and referral pathways for psychosocial support were established in all sites. For the visual methodology, participants were provided with training on key concepts and methods including identification risks for participants.^17 18^ A ‘no faces’ protocol was encouraged; with specific written release forms where identifiable images were deemed to be required.

### Patient and public involvement

Patients and community members were involved as active participants throughout the research process. The study was designed as a participatory prioritisation exercise in which patients/community members, healthcare providers and policymakers were coproducers of findings rather than subjects of observation. Participants defined the research content by photographing aspects of injury care they considered important, developing captions in their own words, and collectively deliberating on and ranking quality priorities. The outcome measures (IoM quality domains) were introduced as a framework, but participants determined local meanings, selected priorities and shaped the interpretation through deliberative dialogue. Participants were involved from the first data collection point (orientation, FGD 1), where their knowledge and experiences informed the direction of the study, through to the prioritisation exercise (FGD 2), where they collectively validated final rankings.

The Photovoice methodology was selected to enable participation by individuals with varying levels of formal education, using visual and narrative approaches rather than solely text-based instruments. The burden of participation was considered in the study design: workshops were limited to two sessions of manageable duration, spaced 7–10 days apart and held at accessible local venues. Participants were reimbursed for travel and time. Dissemination of findings to participants and wider communities is ongoing through the Equi-Injury programme’s stakeholder engagement structures in each country, including feedback workshops with participants and local policymakers. Country-specific summaries of findings were developed for sharing with participant communities in accessible formats, including visual outputs from the Photovoice process.

## Results

**Figure 1:**
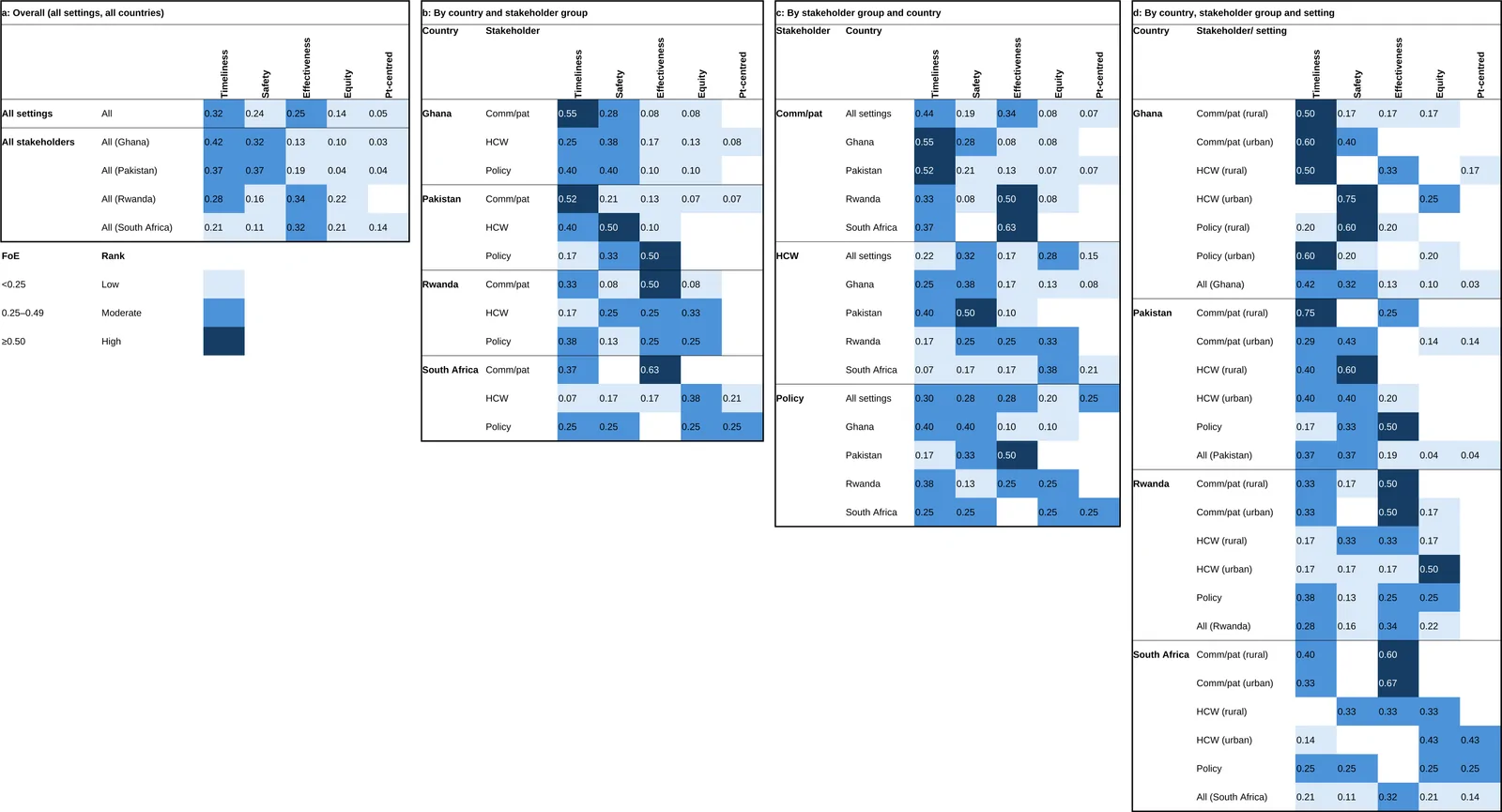
Stakeholder priorities for quality of care: frequency of endorsement (FoE) (0.00–1.00) derived from the prioritisation exercise. HCW, healthcare worker.

The prioritisations revealed observable patterns (figure 1). In Ghana and Pakistan, endorsements clustered around timeliness and safety, reflecting participants’ emphasis on rapid access and avoidance of additional harm across the injury pathway. In Rwanda and South Africa, participants more frequently prioritised effectiveness, which stakeholders predominantly operationalised in terms of functional and psychosocial recovery in addition to clinical care. These contrasts were interpreted alongside characteristics of emergency care systems, including financing and access (table 1).

### Timeliness

Across all settings, timeliness was the most important dimension of quality after injury (endorsement score 0.32). This was prominent in contexts of high system fragmentation, with the highest endorsements in Ghana (0.42) and Pakistan (0.37). Participants discussed timeliness as a whole pathway property beginning ‘from the moment injury happens’ and shaped by first aid, transport, triage, referral and facility processes.

Community members described delays in reaching care: distant facilities, poor roads and reliance on improvised transport. Many described ‘cascades’ of harm, whereby delayed transport or referral reduced the chances of success in subsequent clinical care, contributing to secondary complications (bed sores, infected wounds), escalating costs, prolonged disability and death. Participants consistently described ‘a race against time’, where delays had potentially life-altering consequences (table 2a).

**Table 2:** Visual evidence captions and narrations mapped to IoM quality domains

IoM priority domain	Caption/ photographer	Narration	Ethical rationale for non-reproduction^48^
(a) Timeliness	A broken system: a child’s life lost (community, patients, rural Pakistan)	In a village 20–25 km from the nearest civil hospital, a child was bitten by a cobra. After being transported on a bike loaned by a landlord, the child waited 10–15 min for tests in one village, followed by a 3-hour wait for retesting, only to be told that anti-venom was unavailable. The whole taluka (district) lacked anti-venom, and a 45-minute delay occurred due to the unavailability of an ambulance. Helped by strangers, the child was transported in a random car to an EMS Station 15 km away, where an ambulance refused to assist, and untrained EMTs mishandled the medical needs. Tragically, the child died en-route to the hospital despite the father’s desperate efforts.	The image depicted an unwell infant held in the arms of a partially obscured adult, lacking contextual information. The decision to omit the image was guided by ethical frameworks regarding the visualisation of vulnerability in global health research. In the absence of grounding contextual data, presenting an image of an infant in distress risks objectifying the subject, and limited analytical insight. Visual methodologies demand a high threshold of necessity when displaying minors in healthcare crises to ensure the protection of dignity and avoid reinforcing reductive tropes of infant mortality, helplessness, and suffering. As the image did not offer unique data critical to the study themes, it was not included in the paper to prioritise ethical standards.
(b) Timeliness	Learn something (policymaker, Pakistan)	The ambulance is trapped in a traffic jam caused by improper driving, with cars not following designated lanes and pedestrians crossing the road haphazardly. This standstill puts a trauma patient inside the ambulance at risk of missing the critical ‘golden hour’ for treatment.	The image showed a public road scene with vehicles, road users, and potentially identifiable registration plates and faces. Because the core analytic value lay in the narrated account of congestion and delayed emergency transport, rather than in identities captured in the scene, the image was not reproduced.
(c) Patient centredness	Nurse reassuring a nervous patient (healthcare workers, South Africa)	The patient arrived in the morning for an eye operation appearing nervous, unaware of what was going to be done. On welcoming her, the nurse explained the procedure, reassuring that it would go well. Later, the nurse shared her lunch box considering that the patient left home early that morning. In patient centred care, the relationship between patient and healthcare worker is important as it affects how the patient responds to the healthcare.	The image depicted a clinical interaction between a nurse and a patient in a perioperative context. Reproducing the image would risk compromising confidentiality by making the patient and health worker identifiable within a care encounter. Special caution is warranted where patients are in dependent or vulnerable positions, even when consent has been obtained, because publication may extend exposure beyond what was anticipated. As the key analytic insight concerned reassurance, explanation and relational care, the image was not reproduced.
(d) Equity	Optimal treatment for severely injured (policymaker, Ghana)	This is a picture of a patient who suffered a severe head injury at a mining site receiving optimal treatment including being intubated on a ventilator. This patient was a foreign national alleged to be involved in illegal mining, an action that is currently a big national threat to our drinking water sources with many other documented health implications. Even though he was allegedly involved in an illegal activity for which the government is now arresting all persons involved in it, he still received optimal standard care for his injuries.	The image showed a critically injured patient receiving life-sustaining treatment. The ethical threshold for reproducing images of incapacitated patients in critical care is particularly high, especially where there is potential for legal, social, or political harm. Publication risked identification and possible stigmatisation by linking a severely ill individual to interpretations of illegal activity and foreign nationality. Because the central analytic point was equitable treatment irrespective of social or legal status, the narration was retained but the image was not reproduced.

EMS, Emergency Medical Services; EMT, Emergency Medical Technician; IoM, Institute of Medicine.

Healthcare providers similarly highlighted the ‘golden hour’, referring to delays across care pathways (ambulance dispatch, triage, diagnostic turnaround and inter-facility referral). Health workers linked timeliness to facility readiness (team availability, equipment, handovers), noting delays as frequently compounded by external factors (road congestion). Policymakers framed timeliness as a key performance indicator, also stressing rapid first contact, harmonised referral and emergency transport. The golden hour was again invoked, with delays from congestion and transport highlighted (table 2b).

In all settings, participants noted that delays disproportionately affected poorer, rural and uninsured patients. Overall, participants converged on a view of timeliness as the primary priority and determinant of survival, and that improving it requires attention beyond the emergency department (ED) to prehospital, infrastructural and organisational factors.

### Safety

Safety was also understood as survival-oriented (0.24), with safeguards in on-scene management, transport, triage and treatment. Healthcare providers in Pakistan ranked safety as the top priority (0.50). Safety was operationalised overall as avoiding additional harm throughout the injury pathway and was closely linked to environmental hazards, transport conditions and resource variability.

Communities and patients highlighted unsafe transport (motorbikes, carts, private vehicles without immobilisation), hazardous physical environments (broken bridges, uneven roads) and lay first aid practices that worsened injuries. Photovoice images depicted absent equipment, and contamination hazards in treatment areas.

**Figure 2:**
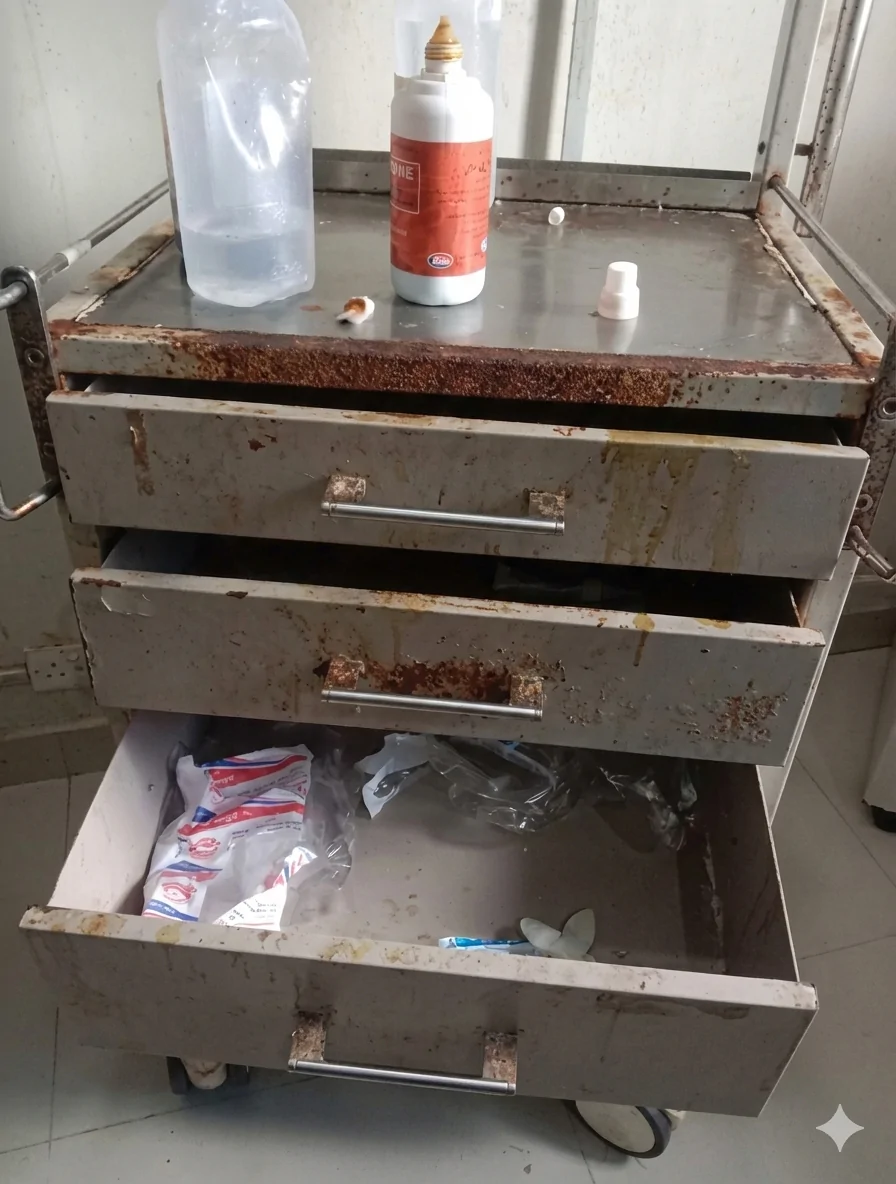
Sterilisation (healthcare worker, Pakistan); IoM priority domain: Safety; Narration: This is a dirty trolley that we use to hold our equipment. We keep instruments on it when doing a procedure, which creates significant risk of infection. Moreover, there are no surgical gloves available.

Healthcare providers understood safety as avoidance of harm arising from institutional hazards (unsterile environments, lack of electricity), describing environments where care processes themselves carried risks. Concerns were expressed over inconsistent injury immobilisation, inadequate infection control and overcrowded EDs that limited patient monitoring (figure 2). They emphasised that inexperienced staff, particularly in rural facilities and resource variability across districts created systematic safety gaps. Ambulance safety, specifically lack of equipment, oxygen and trained staff, was a recurring theme.

Policymakers viewed safety as a governance issue, requiring national standards, staff training and accountability processes. They referenced efforts to standardise trauma protocols, expand emergency care competencies and reduce variability between districts. Policymakers in South Africa highlighted community and gang violence that prevented ambulances from operating safely. Failures in this domain magnified downstream risks, illustrating another ‘cascade’ linking safety to timeliness and effectiveness.

### Effectiveness

Effectiveness was prioritised relatively high overall (0.25). Prioritisation was higher in Rwanda (0.34) and South Africa (0.32) than in Pakistan (0.19) and Ghana (0.13). Community and patient groups in South Africa and Rwanda ranked it especially high (0.63 and 0.50, respectively). Stakeholders interpreted effectiveness broadly, encompassing clinical acuity and recovery with functional and psychosocial rehabilitation, absence of complications and ability to return to work.

Community members and patients highlighted the importance of early management (clean wound care, immobilisation, bleeding control) to prevent chronic disability. Rwandan participants utilised Photovoice images of healed fractures and assistive devices to discuss longer-term outcomes. Photovoice data contrasted properly dressed wounds with chronic injuries attributed to inadequate early care. Participants stressed that ineffective care often began with missed steps in prehospital phases, compounded by early discharge or discontinuity of care due to inability to pay (see Equity, below).

**Figure 3:**
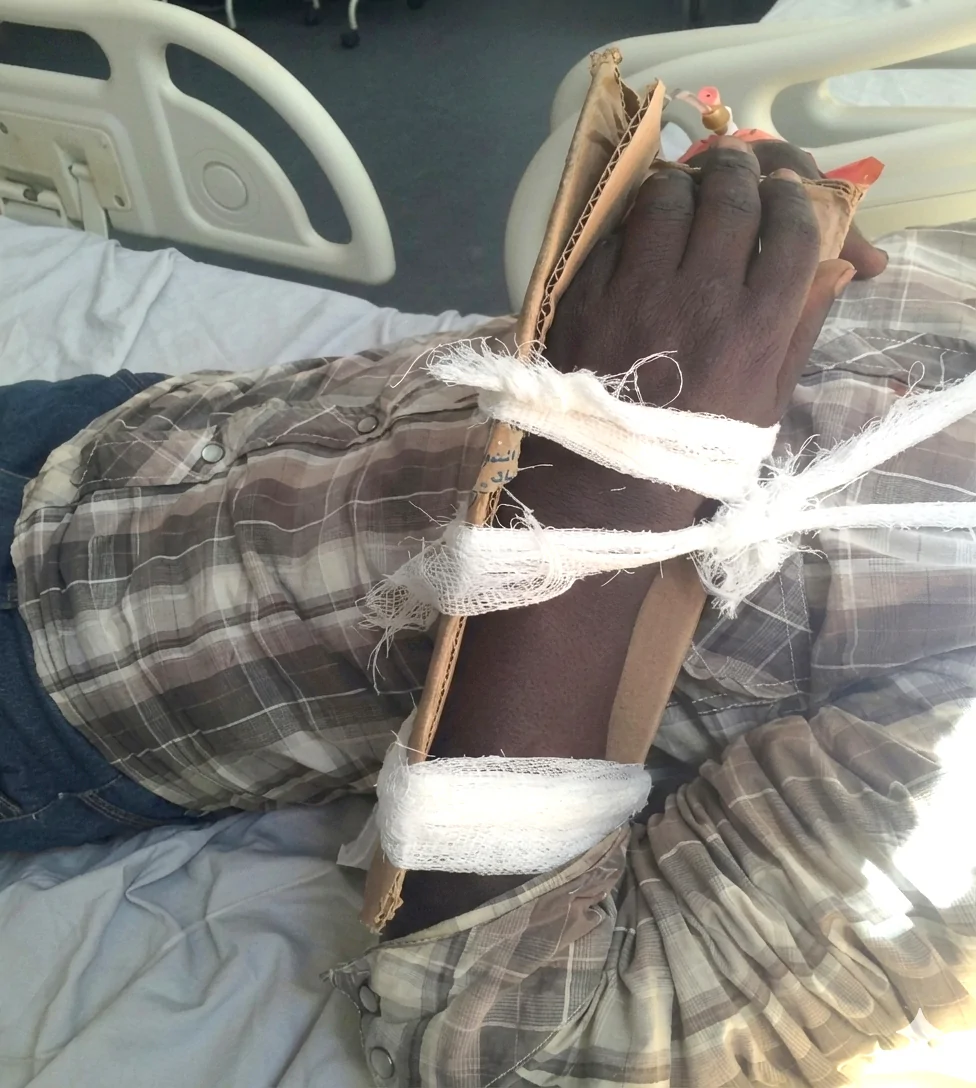
Forearm trauma at health centre (policymaker, Rwanda); IoM priority domain: Effectiveness; Narration: Distal fracture of the right radius. The immobilisation was not done following the standard methods. Even if the health centre used the carton there was inappropriate material to immobilise the fracture. After the X-ray of the wrist, I found that the fracture was not displaced. Therefore, the care received was effective.

Healthcare providers highlighted adherence to protocols, availability of essential and life-saving supplies (traction, antivenom), multidisciplinary teams and clear referral pathways. Providers repeatedly noted shortages of staff or equipment forcing improvisation and compromising outcomes. Policymakers discussed effectiveness in relation to policy guidance and protocols, alongside training and resource allocation, and converging with health workers, acute equipment constraints (figure 3).

### Patient centredness

Patient centredness received lower endorsement scores relative to other domains (0.05), although it was a prominent narrative theme. Participants operationalised patient centredness through relational care features: clear explanations, reassurance, emotional support, dignity, privacy and respectful treatment. Photovoice images depicted clinicians offering comfort and respectful interactions, contrasted with overcrowded spaces that undermined dignity.

An exception to the low overall scoring was among South African stakeholders. Policymakers prioritised patient centredness alongside effectiveness (0.25), viewing it as an integrated systems issue: aligning referral with patient needs, recognising cultural norms, addressing stigma and integrating psychosocial support. Urban healthcare providers similarly viewed the domain as essential for treatment adherence and emotional stability, describing ‘rehumanising practices’ (such as sitting at eye level, explaining procedures in local languages, supporting families) as small actions that restore dignity and trust in high-pressure environments (table 2c). In clinical contexts of high patient load, complex demands and widespread interpersonal violence, staff attitudes were seen as a critical determinant of whether patients would trust or return to the system. Across all contexts, patient-centredness was linked with trust through staff behaviour, service continuity and responsiveness.

### Equity

Equity was prioritised with low frequency across settings (range: 0.04 in Pakistan to 0.22 in Rwanda) and overall (0.14). However, it was referenced persistently. The qualitative analysis clarified that, rather than a competing quality outcome, stakeholders viewed equity as foundational to failures in the other domains. Patients described how lack of money (equity) caused critical delays that led to death (timeliness) or complete exclusion from care (effectiveness). Patients and communities emphasised financial barriers: transport costs, user fees, medicines and investigations causing delays and early discharge. Rural participants in all countries highlighted longer distances, fewer ambulances, weaker facility capacity and slower referral. Individuals from minority or disadvantaged groups also described feeling deprioritised or disrespected.

Health workers and policymakers across settings ranked equity higher than communities and patients. Providers highlighted geographic disparities in equipment and staffing, emphasising that poorer facilities shouldered greater injury burdens with fewer resources. Policymakers acknowledged inequities were produced by insurance verification processes and fragmented public–private configurations. Many noted national protocols as tools to promote fairness (table 2d). Overall, providers and policymakers more often articulated allocative and systems wide inequalities, whereas patients and communities selected domains reflecting proximal, experiential barriers such as transport, user fees and early discharge.

This divergence has implications for how quality is defined and measured. Where equity is assessed solely through systems level indicators such as geographic coverage or insurance enrolment, the experiential barriers that most directly shaped patient outcomes identified in this study (affordability of transport, OOP costs and ability to remain in care) risk being overlooked.

## Discussion

Across four LMIC settings, stakeholders articulated quality after injury as a cumulative, relational property of the care pathway rather than a set of independent domains operating in isolation. This challenges the way quality frameworks are operationalised in parallel dimensions for measurement and reporting, and suggests that for injury care, domains function interdependently, with failures propagating across the pathway as ‘cascades of harm.’

The dominant priority was timeliness, framed not as waiting room time but as a whole pathway property from injury occurrence to definitive care. This aligns with the Three and Four Delays frameworks^19–21^ and with delays identified in Equi-Injury’s cohort work.^22^ This study adds empirical evidence, generated by stakeholders themselves, on the mechanisms through which delays cascade. These included transport failures, compounding triage delays, compounding clinical outcomes and visible through Photovoice in ways not captured by conventional survey or facility data. The broad consensus on timeliness across all stakeholder groups and settings suggests prehospital care may represent a ‘united goal’ capable of supporting community and political buy-in for reform.

Where basic emergency access was more established, Rwanda^23^ and South Africa,^24^ (table 1), priorities shifted towards effectiveness, defined broadly to encompass functional rehabilitation, psychosocial recovery and return to work, and not only clinical management. Community and patient groups in these settings endorsed effectiveness at notably high levels (0.50 and 0.63, respectively). This underscores a gap in injury care quality monitoring: long-term functional outcomes and patient-reported recovery are not consistently captured in LMIC trauma registries.^4 5 25 26^

Safety, ranked highest by providers in Sindh, Pakistan (0.50), was defined in terms beyond clinic error, by the absence of basic protections: unsafe transport, unsterile environments, lack of electricity and resource variability across districts. This supports evidence that safety failures in LMICs are predominantly environmental and systemic^24 27^ requiring attention across prehospital systems, training, infrastructure and governance rather than individual-level error reduction.

Patient centredness scored low quantitatively but was salient qualitatively, particularly in South Africa where providers described ‘rehumanising practices’ as small discretionary actions with significant impacts restoring dignity in high-pressure environments and circumstances. This finding extends literature showing that communication and relational quality influence adherence, satisfaction and willingness to seek care,^4 28^ and suggests that in contexts of interpersonal violence and high patient load, trust may function as a precondition for effective care.

An analytically distinctive finding was on equity. Despite low endorsement as a discrete domain (0.14), equity was consistently positioned as foundational, as an upstream condition that shaped other domains, particularly timeliness and effectiveness, rather than as a discrete quality outcome to be ranked against them. In many accounts, lack of money, insurance gaps and rural–urban differences translated into delayed care seeking, disrupted referral, early discharge and catastrophic costs, with downstream consequences. This has implications for how quality is measured. Where equity is assessed through systems level indicators such as geographic coverage or insurance enrolment, the experiential barriers that directly shaped outcomes in this study such as affordability of transport, OOP costs, ability to remain in care, risk being overlooked. The divergence between stakeholder groups reinforces this: providers and policymakers articulated allocative and structural inequalities, whereas patients described proximal, experiential barriers. Quality frameworks that do not incorporate both perspectives may systematically undercount barriers that are significant to those navigating the system.

The ‘cascades of harm’ made visible through Photovoice extend existing frameworks by showing how stakeholders themselves trace failures across the care pathway, as lived sequences with cumulative consequences for survival, disability and impoverishment rather than abstract concepts or components. This aligns with the WHO Emergency Care System Framework,^27 29^ the WHO Integrated People-Centred Health Services model,^30^ OECD models^31^ and the Lancet Commission on High Quality Health Systems,^4^ which highlight integrated pathway approaches but have lacked empirical evidence of how stakeholders in LMICs experience and prioritise these dynamics.

Overall, quality was understood as a cumulative and relational property of the injury pathway, with variations by systems capabilities. In Rwanda and South Africa, prioritisation of effectiveness (0.34 and 0.32, respectively) suggests that when survival is more probable, attention turns to recovery and quality of life. In Ghana and Pakistan, priorities clustered around domains critical for immediate survival, timeliness and safety. These patterns were interpreted alongside contextual characteristics and are presented as emerging from the data rather than a formal health system classification.

The implications for policy and practice are as follows. First, the consensus on prehospital care potentially represents a ‘united goal’ supporting community buy-in. First response, safe transport, referral and facility readiness should be integrated as a single reform package (including minimum standards for triage, infection prevention, equipment and staffing).^19 32^ Second, trauma quality indicators should be extended beyond facility processes to capture what stakeholders value including time to definitive care and effectiveness inclusive of recovery (functional and psychosocial outcomes, rehabilitation/assistive devices, return to work).^33^ Aligning metrics with stakeholder priorities can strengthen both accountability and shared advocacy. Third, equity should be treated as an enabler of quality rather than a parallel objective. The implications include reducing catastrophic costs (transport, diagnostics, medicines, postacute care) and ensuring that quality governance includes the perspectives of those who experience these barriers. The divergence in how stakeholder groups understood equity underscores the need for local quality committees with strong community representation.^34^ Community governance bodies are well-established in PHC^35 36^ and may have direct applicability for injury care. It should be noted that the four countries differ substantially, and attributing priority differences to specific system features requires caution.

The study had several strengths. The comparative multicountry design and multimethod approach enabled cross-site analysis while preserving contextual depth. Photovoice enabled stakeholders to show, explain and visualise relational dynamics, and the deliberative prioritisation process ensured that rankings were collectively validated rather than individually assigned. There were several limitations. Participants were engaged through facility-linked and established stakeholder groups, introducing potential selection bias toward those already connected to formal health systems. Convening policymakers proved logistically challenging,^37^ and in Pakistan interviews replaced group deliberation. Participation in Photovoice required access to a camera-enabled device and the ability to use it. Where needed, the research team provided brief practical supports; however, limited device access may have constrained participation for some individuals. While participants were very clear on the meanings of the visual data, articulating clear accounts of photographs was challenging and some nuance may have been lost in the process. Also, while priorities were implicit in the deliberations, preference weights were not formally quantified, and so a domain endorsed by fewer participants may still have been their highest priority. Omission of the efficiency domain also limited explicit capturing of resource and waste issues. These issues were nevertheless frequently articulated through other domains (resource scarcity, unsafe conditions associated with shortages).

The IoM domain labels are anglophone, while data collection across study settings was conducted in local languages: Twi, Dagbani and English in Ghana; Urdu and Sindhi in Pakistan; Kinyarwanda in Rwanda and isiXhosa, Afrikaans and English in South Africa (with arrangements varying by zone and stakeholder group). Recordings were translated into English for coding, and we did not undertake a formal mother-tongue sensitivity analysis. Several considerations are noteworthy in this regard. First, local meanings were brought into the analysis through facilitator-led deliberation, with participants encouraged to articulate priorities in their own terms before these were mapped onto the IoM domains. Cross-language interpretation may nevertheless have shaped how participants articulated and prioritised quality domains despite collaborative local facilitation and analysis. Second, RREAL sheets captured procedural reflections at the point of data collection, including translation issues. Third, cross-linguistic consistency of domain interpretations (eg, safety being construed across settings as environmental and structural rather than solely in terms of clinical error) may reflect both genuine convergence in stakeholder framings and translation constraints, which we cannot fully separate analytically. Future Photovoice work would benefit from in-language coding and explicit cross-linguistic comparison of domain semantics, particularly for terms such as safety, patient-centredness and equity, which carry distinct connotations across linguistic and cultural contexts.

## Conclusions

How patients, providers and policymakers experience quality across injury care pathways remains largely absent from efforts to define and measure it. In our study across four LMICs, participant groups articulated a coherent, systems level understanding of quality after injury, framing it as cumulative across the care pathway rather than as a set of discrete domains. Priorities converged on timeliness, safety and effectiveness but were shaped by local system capability and stakeholder perspectives. Policymakers emphasised standards, performance and governance. Providers prioritised reliable resources, staffing and safe working conditions. Patients and communities defined quality through affordability, transport access, respect and freedom from catastrophic costs. Equity was positioned as foundational across all groups. These priorities are currently largely absent from efforts to define and measure injury care quality and should be integrated into reforms linking prehospital care, facility readiness, financial protection and functional recovery to build trauma systems that are responsive to the contexts and people they serve.

## Supplementary material

10.1136/bmjgh-2026-024260 online supplemental file 1

10.1136/bmjgh-2026-024260 online supplemental file 2

## Data Availability

Data are available on reasonable request.
