# Supplementary material for: ‘A race against time’: a photovoice study on priorities for quality of injury care with patients, health workers and policymakers in four low- and middle-income countries
Source: BMJ Glob Health. 2026 Aug 20;11(8):e024260. doi: 10.1136/bmjgh-2026-024260 (PMC13504915; doi:10.1136/bmjgh-2026-024260)
Supplement: Supplementary file 1 [file bmjgh-11-8-s001.docx]

### BMJ Global Health Author Reflexivity Statement

Adapted from Morton, B., Vercueil, A., Masekela, R., Heinz, E., Reimer, L., Saleh, S., Kalinga, C., Seekles, M., Biccard, B., Chakaya, J., Abimbola, S., Obasi, A. and Oriyo, N. (2022), Consensus statement on measures to promote equitable authorship in the publication of research from international partnerships. Anaesthesia, 77: 264-276. <https://doi.org/10.1111/anae.15597>

| **Study conceptualisation** | |
| --- | --- |
| 1. How does this study address local research and policy priorities? | Injuries are a leading cause of death and disability in all four study countries, and improving quality of injury care is a recognised health system priority in each setting. The study directly addresses local policy needs by identifying how patients, frontline providers, and policymakers themselves prioritise quality of care. These perspectives are largely absent from existing frameworks. The research was embedded within the NIHR Equi-Injury programme, which was developed in response to locally identified gaps in injury care governance, access, and quality across Ghana, Pakistan, Rwanda, and South Africa. Findings are intended to inform context-specific quality improvement strategies and have been shared with national and local policymakers through programme stakeholder structures in each country. |
| 2. How were local researchers involved in study design? | Local principal investigators in each country (Ghana: ST, FSA; Pakistan: JR, ZWA; Rwanda: AB, JCB, DFB; South Africa: KC, NT) were involved in the conceptualisation and design of the Equi-Injury programme from its inception, including the development of the published study protocol. The Photovoice and deliberative prioritisation methodology was co-designed with country teams, who adapted data collection instruments, topic guides, and facilitation approaches to local languages, cultural norms, and health system contexts. Local teams determined site selection and recruitment strategies appropriate to their settings. |
| **Research management** | |
| 3. How has funding been used to support the local research team(s)? | NIHR programme funding was distributed across all four country partners, supporting local researcher salaries, fieldwork costs, participant reimbursements, equipment, and travel for data collection across urban and rural sites. Funding also supported training workshops in Photovoice methodology, qualitative analysis, and academic writing for local team members, including early career researchers. |
| **Data acquisition and analysis** | |
| 4. How are research staff who conducted data collection acknowledged? | All research staff who made substantive contributions to data collection are named as co-authors on this manuscript in accordance with ICMJE criteria, with contributions documented using the CRediT taxonomy. Local researchers who facilitated focus group discussions, conducted interviews, managed transcription and translation, and coordinated fieldwork across all eight sites are included in the author list. The manuscript is published under the Equi-Injury Group, reflecting the collaborative and distributed nature of the work. |
| 5. How have members of the research partnership been provided with access to study data? | All country teams have full access to the data collected in their respective settings, including audio recordings, transcripts, photographs, and RREAL sheets. Translated transcripts and the shared NVivo coding framework are accessible to all analytical team members across the partnership. Data ownership is shared, with country teams retaining the right to use their data for additional analyses and publications. |
| 6. How were data used to develop analytical skills within the partnership? | Qualitative analysis was conducted collaboratively, with local researchers leading the coding and interpretation of data from their own contexts. Cross-site analytical workshops were held to develop the shared coding matrix, discuss emergent themes, and build qualitative research skills across the partnership. Early career researchers in all four countries participated in data analysis and were supported by senior team members in developing proficiency in NVivo, thematic analysis, and the frequency of endorsement methodology. |
| **Data interpretation** | |
| 7. How have research partners collaborated in interpreting study data? | Interpretation was an explicitly collaborative process. Country teams led interpretation of their own data, ensuring contextual accuracy and relevance. Cross-country interpretation sessions were held to compare patterns, discuss divergences, and develop the overarching analytical framework. All authors reviewed and contributed to the interpretation presented in the manuscript. The deliberative Photovoice methodology itself supported collaborative interpretation, with study participants selecting, narrating, and collectively prioritising findings before researcher-led analysis. |
| **Drafting and revising for intellectual content** | |
| 8. How were research partners supported to develop writing skills? | The Equi-Injury programme includes structured support for academic writing across the partnership. Writing workshops were held for early career researchers, covering manuscript structure, argumentation, and publication processes. Drafts of this manuscript were developed and integrated by the lead authors, with rounds of iterative feedback with partners provided to support skill development. Early career researchers from LMIC partner institutions have led or co-led publications from the wider Equi-Injury programme. The programme's publication strategy explicitly prioritises LMIC researcher leadership on outputs arising from country-specific and comparative analyses. |
| 9. How will research products be shared to address local needs? | Findings have been and will continue to be shared through programme stakeholder engagement structures in each country, including dissemination with policymakers, health system managers, and community representatives. The manuscript will be published open access to ensure accessibility in all study countries. Visual outputs from the Photovoice process (photographs and narratives) have been shared with participants and local stakeholders. Country-specific policy briefs summarising priorities and actionable recommendations are planned for dissemination to national and district-level decision-makers. |
| **Authorship** | |
| 10. How is the leadership, contribution and ownership of this work by LMIC researchers recognised within the authorship? | Of the authors group, the majority are based in institutions in the four LMIC study countries. Local principal investigators from all four countries contributed to conceptualisation, funding acquisition, investigation, and supervision, and hold authorship positions reflecting these contributions. Two LMIC-based authors (NT, DFB) share early authorship positions. Senior LMIC investigators (ST, AB, JCB, JR, KC) hold authorship positions commensurate with their leadership roles in the programme. |
| 11. How have early career researchers across the partnership been included within the authorship team? | Early career researchers from all four study countries are included as authors in recognition of their substantive contributions to data collection, facilitation, analysis, and interpretation. These include researchers at doctoral and postdoctoral level from University for Development Studies (Ghana), Aga Khan University (Pakistan), University of Global Health Equity (Rwanda), and Stellenbosch University (South Africa). The programme has actively supported early career researchers to take on increasing responsibility across study phases, with a view to their leading future publications from the programme. |
| 12. How has gender balance been addressed within the authorship? | The author team comprises 18 women and 19 men (37 authors). Gender balance was actively considered in the composition of country research teams and in authorship decisions. Women hold key leadership positions in the partnership, including corresponding author and equal first author (LD, AI), and country lead roles (NT, ZWA, AEA). The senior author (JD) is also a woman. |
| **Training** | |
| 13. How has the project contributed to training of LMIC researchers? | The programme has provided training in Photovoice methodology, qualitative and mixed-methods research, ethical conduct of visual research, academic writing, and stakeholder engagement. Training was delivered through in-country workshops, cross-programme meetings, and mentorship from senior investigators. Early career researchers have developed skills in facilitation, qualitative coding, cross-site comparative analysis, and manuscript preparation. |
| **Infrastructure** | |
| 14. How has the project contributed to improvements in local infrastructure? | The project has strengthened local research infrastructure by establishing stakeholder engagement platforms in each country that bring together patients, providers, and policymakers around injury care quality. These platforms extend beyond the research programme and have potential for sustained use in quality improvement governance. The programme has also contributed to local capacity for visual and participatory research methodologies that remain with local teams. |
| **Governance** | |
| 15. What safeguarding procedures were used to protect local study participants and researchers? | Ethical approval was obtained from institutional review boards in all four countries. Written informed consent was obtained from all participants, with separate consent for photographic subjects where necessary. A 'no faces' protocol was encouraged to protect participant identity, with written release forms required where identifiable images were deemed necessary. Facilitators monitored participant wellbeing during data collection, and referral pathways for psychosocial support were established at all sites. Participants were trained on risks related to identification in visual research. The deliberative methodology ensured participants retained control over image selection, narration, and final priority rankings. Researcher safety protocols were established for fieldwork across all urban and rural sites. |
