# Supplementary material for: ‘A race against time’: a photovoice study on priorities for quality of injury care with patients, health workers and policymakers in four low- and middle-income countries
Source: BMJ Glob Health. 2026 Aug 20;11(8):e024260. doi: 10.1136/bmjgh-2026-024260 (PMC13504915; doi:10.1136/bmjgh-2026-024260)
Supplement: Supplementary file 2 [file bmjgh-11-8-s002.docx]

Supplementary material 1. Institute of Medicine (IoM) quality domains: definitions and interpretations for injury care summarising how participants operationalised domains during deliberation

| Domain | Definition | Interpretations for injury care |
| --- | --- | --- |
| Timeliness | Reducing waits and sometimes harmful delays for both those who receive and those who give care. | Operationalised as a whole pathway property, from injury occurrence to definitive care and encompassing first aid, transport, referral and within facility processes, not only transfers or waiting room time. |
| Safety | Avoiding harm to patients from the care that is intended to help them. | Emphasised safeguarding patients across the injury pathway (scene, transport, triage and treatment), including preventing iatrogenic harm, infection, and hazards arising from resource constraints. |
| Effectiveness | Providing services based on scientific knowledge to all who could benefit and refraining from providing services to those not likely to benefit (avoiding underuse and overuse). | Operationalised in outcome-oriented terms, including functional recovery and psychosocial rehabilitation (e.g., ability to return to work), alongside correct clinical management and complication prevention. |
| Equity | Providing care that does not vary in quality because of personal characteristics such as gender, ethnicity, geographic location, and socioeconomic status. | Commonly discussed through financial and geographic barriers (out-of-pocket costs, insurance status, rural-urban access) and how they shaped entry to care and treatment. |
| Patient centredness | Providing care that is respectful of and responsive to individual patient preferences, needs, and values, and ensuring that patient values guide all clinical decisions. | Emphasised communication, dignity and trust, being informed, reassured and respected, alongside privacy and responsiveness to patient needs and values. |
| Efficiency | Avoiding waste, including waste of equipment, supplies, ideas, and energy. | Efficiency was excluded from the prioritisation exercise in line with the parent study protocol, which considered efficiency beyond the remit of this study (8). |
